# Moving to Serene Nature May Prevent Poor Mental Health—Results from a Swedish Longitudinal Cohort Study

**DOI:** 10.3390/ijerph120707974

**Published:** 2015-07-14

**Authors:** Matilda Annerstedt van den Bosch, Per-Olof Östergren, Patrik Grahn, Erik Skärbäck, Peter Währborg

**Affiliations:** 1 Faculty of Landscape Architecture, Horticulture and Crop Production Science, Swedish University of Agricultural Sciences, S-23053 Alnarp, Sweden; E-Mails: patrik.grahn@slu.se (P.G.); erik.skarback@slu.se (E.S.); peter@wahrborg.se (P.W.); 2 Department of Community Health, Malmö University Hospital, Lund University, S-20502 Malmö, Sweden; E-Mail: per-olof.ostergren@med.lu.se

**Keywords:** longitudinal, mental health, nature type, public health survey, recreation, GIS, wellbeing, salutogenic

## Abstract

Green spaces are recognized for improving mental health, but what particular kind of nature is required is yet not elucidated. This study explores the effect of specific types of recreational nature qualities on mental health. Longitudinal data (1999/2000 and 2005) from a public health survey was distributed to a stratified sample (*n* = 24,945) of a Swedish population. People from rural or suburban areas (*n* = 9230) who had moved between baseline and follow-up (*n* = 1419) were studied. Individual geographic residence codes were linked to five predefined nature qualities, classified in geographic information systems (GIS). Any change in the amount of or type of qualities within 300 m distance between baseline and follow-up was correlated to any change in mental health (as measured by the General Health Questionnaire) by logistic regression models. On average, the population had limited access to nature qualities both pre- and post-move. There was no significant correlation between change in the amount of qualities and change in mental health. However, the specific quality “serene” was a significant determinant with a significantly decreased risk for women of change to mental ill-health at follow-up. The objective definition of the potentially health-promoting quality may facilitate implication in landscape practice and healthy planning.

## 1. Introduction

Despite the fact that the health situation has in general become better, and longevity is increasing in Sweden, growing problems with mental disorders are reported. According to official statistics (2011–2014) from the Swedish Public Health Authority [1] the prevalence of impaired mental wellbeing is 18%; whereof 14% among men and 23% among women in southern Sweden. For the entire country corresponding figures are 17%, and 14% and 20% respectively. The most common diagnoses are stress related states (e.g., depression, anxiety and burnout disorders) and the prevalence as well as increase of mental illness are higher among women [2,3]. This pattern is mirrored internationally and unipolar depressive disorders are now the leading cause of disability in middle and high income countries [4].

The aetiology of non-communicable diseases (NCDs) such as mental and cardiovascular disorders, is typically multifactorial. Causal relationships are difficult to define as genetic, psychological, environmental, and social health determinants must all be accounted for. Apart from risk factors for disease, the inverted relation of health factors requires attention from a public health perspective, where health promotion is a core topic. Factors that improve health are similarly multiple and interactive, and follow-up studies are needed to suggest causality.

A salutogenic approach to health encourages health promotion by focusing on health factors rather than risk factors [5]. One such health factor that has lately gained attention is access to nature [6]. Research on health effects of green, natural environments has demonstrated improved psychological wellbeing, reduced stress levels, reduced mortality in cardiovascular diseases, and decreased socioeconomic health inequities [7,8,9,10,11]. In addition, access to green environments has positive effects on physical activity and obesity [12,13,14].

Various theories and pathways have been proposed for explaining the positive relation between green environments and health. Theories suggest for example that humans have an innate preference for natural environments, based on our evolutionary need for nature elements, such as fresh water and the shade of trees [15]. This would in turn contribute to automatic stress reduction in such landscapes [16]. Other theories have stressed the potential for natural environments to provide attention restoration by nature’s optimal balance between input and demands [17]. Parts of these theories have gained empirical support [18,19]. Other research has demonstrated that particular patterns of nature or sounds of nature can have positive effects [20,21].

### Recreational Nature Qualities and Geographic Information Systems

There is a lack of knowledge about which particular types and qualities of nature are most beneficial to health. However, the issue has been approached by a few attempts to defining nature qualities. Extensive research by social landscape expertise, including field studies, interviews and inventories, has resulted in a definition of eight recreational nature qualities, which are important for stress relief and wellbeing [22]. The qualities were derived from an initial amount of more than 50 experiences described in a large amount of interviews performed with people in urban green spaces between 1985 and 2012 [22,23,24,25,26,27]. Using statistical factor analysis the final eight characteristics of nature qualities were identified. The qualities are termed Serene, Wild, Lush, Space, the Common, the Pleasure garden, Festive, and Culture [25], and represent people’s perceived sensory dimensions of varied nature qualities. Several municipalities in Sweden have used the qualities to identify recreational areas on a local level and they have been implemented as indicators for environmental impact assessment in landscape and urban planning [24]. The qualities have at several occasions been re-evaluated and the names have been adjusted [26,27], but the core concepts and definitions remain the same. This article is based mainly on the original terms.

In order to make an attempt of objectifying the nature qualities the characteristics were inventoried and classified outdoors in fields by landscape researchers. This can be described as a first intuitive and discerning classification method. Secondly, this inventory classification was compared with specific maps. Different combinations of variables were tested and compared with the inventory classification. When the correspondence between the GIS maps and the inventory classification was considered satisfactory, the final combination of GIS variables for classifying each characteristic was applied [28,29]. This approach for identification implies that several objective landscape indicators may apply to more than one nature quality and the qualities may to some extent overlap (see Figure 3). The mapping of the qualities was conducted through The National Land Survey of Sweden (Lantmäteriet). Within the European Union programme, CORINE (Coordination of Information on the Environment) Lantmäteriet has mapped and classified the land and vegetation cover of Sweden into 58 land use units, using 25 × 25 m grids [29]. This national land use database in combination with a regional GIS database provided information detailed enough for defining the qualities with land use data and to store them in the GIS-layers. Additional data sources that have been used for classifying specific features, such as noise, serenity, and culture, are for example Natura 2000 (from the Swedish Environmental Protection Agency), demographic data together with traffic data, acoustic data from the County Administrative Board (Länsstyrelsen), detailed data from municipalities, and topographic data.

**Figure 1:**
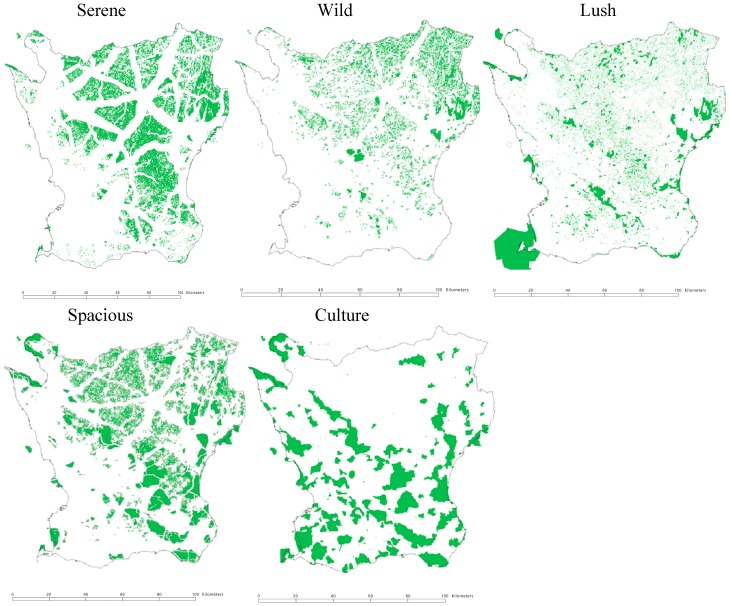
Map over Southern Sweden (county Scania) showing the distribution of five of the nature qualities: Serene, Wild, Lush, Spacious, and Culture.

**Figure 2:**
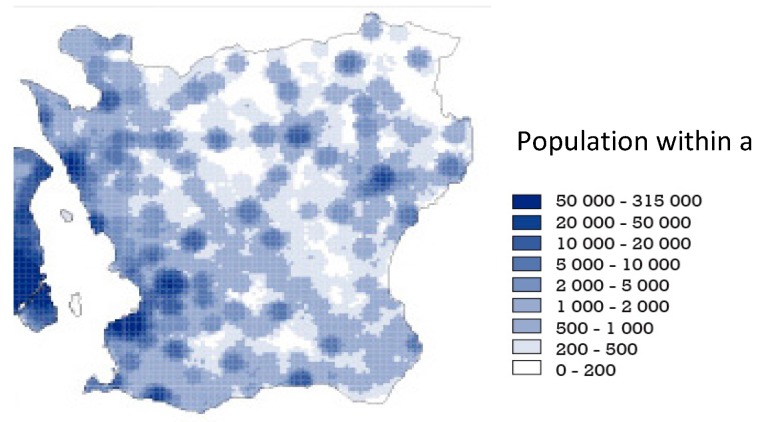
Map of Southern Sweden (county Scania) showing population density across the region. © Lantmäteriet (License No. I2014/00764).

In previous studies these GIS-defined qualities have been associated to neighborhood satisfaction, physical activity, and general and mental health [12,30,31]. The GIS-definitions have enabled the use of the qualities for planning on a regional level [32] and the method has been exported internationally [33,34,35]. The qualities were used as gold standard in a study, where area-aggregated assessments of perceived qualities of green neighborhood demonstrated convergent as well as concurrent validity [36]. The qualities have continuously been validated through repeated field studies and projects [37]. Figure 1 demonstrates the localization, overlap, and distribution of the five qualities that were used in this paper on a map of southern Sweden (Scania). Figure 2 shows the population distribution.

Due to a rising number of individuals suffering from mental diseases and the potentially preventive effect of certain nature qualities, the use of green spaces in public health actions should be optimized. Data from the public health survey used in this article have been published in previous papers [31,37]. However, in this article we wanted to study particularly the group that had moved between baseline and follow-up in combination with the objectively GIS-defined nature qualities, and specifically include investigation of each quality respectively. This has not been studied before and the results could potentially have a specific impact for landscape planning, by providing advice on which quality or qualities should be considered for optimal mental health promotion. As the qualities are defined with land use data the advice would be practically applicable from a planning perspective.

The overall aim of the present study was to investigate changes in mental health related to changes in access to nature qualities in the neighborhood, as an effect of moving to another area. This study does not investigate quantity of green space, but focuses on recreational nature qualities and diversity of green space.

Our specific research questions were:

1. Among those that have moved in the cohort—is there a correlation between change in access to nature qualities in the neighborhood and change in mental health?
2. Do any of the nature qualities demonstrate a higher mental health impact than the others?
3. Do the known gender differences in mental health display in the above postulated associations?

## 2. Experimental Section

### 2.1. Study Population

We analyzed data from a follow-up public health study in a population from southern Sweden. The Swedish registration system provides a personal identification number for every individual, which can be used to link data from different registers, and can be used to follow each individual during the entire study period. The individuals, aged 18–80 years, were followed from 1999/2000 to 2005. A health survey was distributed as a mailed questionnaire in 33 municipalities in southern Sweden. The total sample comprised 24,945 persons. Three mailed reminders and one reminder by telephone were used. At baseline, answers were obtained from 13,604 (54.5%) respondents and 10,485 (77%) responded at follow-up. In this study, we excluded individuals from the larger city centers of Malmö, Lund, Kristianstad, and Helsingborg (*n* = 1245) due to insufficiently detailed urban land use data for making the GIS-definition of the qualities possible in these areas. The final cohort included 9230 persons.

Initially, the survey was stratified to constitute a representative of the total population regarding gender, age, and education level [38]. At follow-up, women were slightly overrepresented (55.4% *vs.* 49.7% of non-participants) and fewer persons were born outside Sweden (9.2% *vs.* 14.8% among non-participants). There were also differences regarding unemployment (4.7% *vs.* 9.5% among non-participants), students (3.4% *vs.* 17.1% among non-participants), and non-manual employees at higher, medium, or lower level (10.6%; 17.6% resp. 15.5% *vs.* 9.1%; 11.0% resp. 8.5% among non-participants).

Extreme values (“outliers”) at follow-up were controlled for and replaced with the values from the survey in 1999/2000. This was done for 60 cases concerning tallness, 10 cases concerning age, and two cases concerning weight and ‘number of persons in the household’ respectively.

### 2.2. Questionnaires

The survey contained in total 106 questions on varied aspects of health and demographic details. Mental health was measured by the General Health Questionnaire (GHQ-12). The GHQ-12 is a shortened 12-item version of the GHQ-28 [39], and is among the most widely used screening instruments for mental disorders with results comparable to those of longer versions of GHQ [40]. Prevalence of poor mental health is defined as reporting a problem to three or more of 12 questions in the GHQ-12 [41]. Each item (e.g., *Have you, during the past few weeks, felt unhappy and depressed*) is rated on a four-point Likert scale: (1) less than usual; (2) no more than usual; (3) more than usual; (4) much more than usual. Reporting problem is defined as rating 3 or 4 on the item (scoring 0-0-1-1). GHQ-12 has internationally demonstrated high validity and reliability [42]. The effects of age, gender, and education level on the screening performance of GHQ-12 have proven to be non-significant [42].

In this study, GHQ-12 was used in Swedish and all items were applied. We used GHQ-12 as a measure to determine any changes in general mental ill health. The study was conducted in accordance with the Declaration of Helsinki and approved by the local committee of ethics.

### 2.3. Land Use Data and Nature Qualities

**Figure 3:**
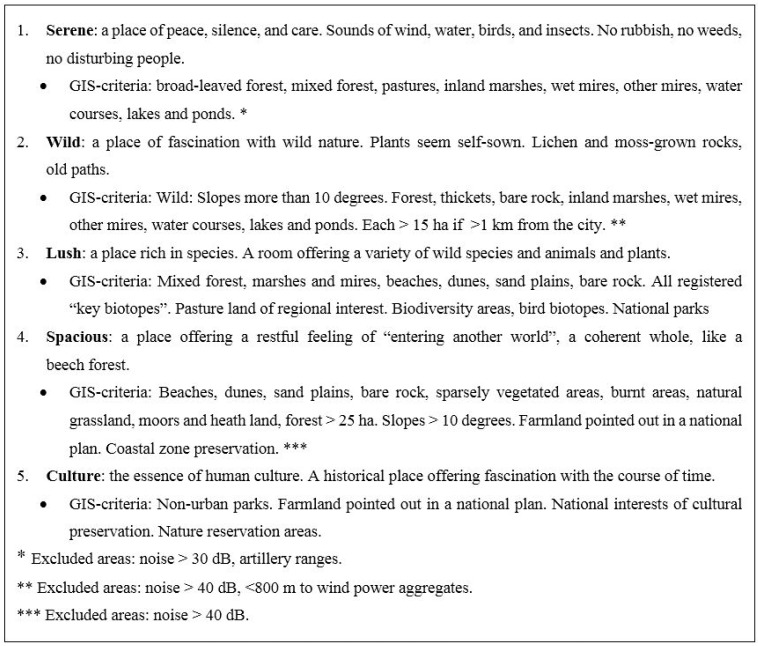
Description of the perceived sensory dimensions of the recreational nature qualities and corresponding GIS-definitions. The decibel criteria only relates to disturbing noise, such as traffic, but not absolute sounds levels from, for example, twittering birds and other nature sounds.

Currently available land use data did not permit objective assessment of the three qualities the Common, the Pleasure garden, and Festive. The remaining five qualities (Serene, Wild, Lush, Spacious, and Culture) were defined with land use data as outlined in Figure 3. Residential geocodes were obtained for each participant and a population GIS-layer was created and combined with the layer of nature qualities. Buffer zones of 300 m (assumed to represent a suitable walking distance [43,44]) were created around each participant and analyzed for existing qualities. This means that if, for example, the area was covered with the quality Serene, but no other qualities, the score would be 1, while as for another area with less total quantity of green space, but containing for example both Serene, Lush, and Spacious, the score would be 3.

### 2.4. Statistical Analysis

According to the validated syntax for GHQ-12 a binary value (good or poor mental health) was calculated for each individual at baseline and follow-up. We analyzed the immediate relationship between change in recreational environment and change in mental health, the trigger effect, between baseline and follow-up among the cases that had moved (*n* = 1419) and had experienced a change in amount of or type of nature qualities. The analyses were also performed separately for those who did not move, and on the full material. Paired samples *t*-tests or ANOVA were performed for measuring any difference between groups (group “movers”, group “remainers”, or group “all”) and between baseline and follow-up.

Two binary delta-values were constructed to identify the participants that had experienced a *change* in mental health: (1) “remedy” (poor mental health at baseline and good at follow-up); and (2) “sicken” (good mental health at baseline and poor at follow-up). At follow-up, each participant could be one of the four alternatives: (1) “remedy”; (2) “sicken”; (3) “remain good”; or (4) “remain poor”, but in the logistic regression only those who had perceived a change in mental health (“remedy” or “sicken”) were included.

Change in mental health between baseline and follow-up was cross correlated (by Spearman’s correlation coefficient) with change in number of nature qualities within 300 m or change in types of nature quality separately. By logistic regressions we studied correlations between change in amount of or change in types of nature qualities in the neighborhood, and change in mental health, while adjusting for individual age, ethnicity, perceived stress, education level, and economy. Perceived stress was measured by a single item question relating to experience of daily life stress rated on a four point Likert scale from never to often. The confounders were retained in the model only if they reached a significance level below 0.2 [45,46]. All analyses were conducted using SPSS 18.0 for Windows (SPSS Inc., Chicago, IL, USA). Statistical significance level was set to *p*-value < 0.05 and 95% confidence intervals (CI) for mean differences and odds ratios (OR).

## 3. Results

The prevalence of poor mental health was 16.4% and 15.8% at baseline and follow-up respectively in the whole material. In the group that moved, the prevalence was 21.0% and 20.6% respectively (see Table 1). In the whole material 9.8% and 9.3% belonged to respectively “remedy” and “sicken”, *i.e.*, had changed from bad to good mental health and vice versa. Among the “movers” the corresponding figures were 11.6% and 11.4% respectively. Thus, among the “movers” around 21% reported poor mental health at both occasions, but about 11% had experienced a change in either a good or a bad direction. Even though there is no change in the absolute amount of individuals with poor mental health we know there is a change of individuals.

There were no significant differences in amount of nature qualities or in mental health between baseline and follow-up in either of the groups and the cross-correlations (Spearman’s correlation coefficient) were non-significant. The “movers” belonged to a significantly higher education group, were significantly younger, had significantly more nature qualities, and had significantly poorer mental health (both occasions), compared to the “remainers”. No gender difference was seen between the groups.

The most common quality in the neighborhood among “movers” was Culture. Among “remainers” and “all”, Lush was most abundant. Wild was least common and Serene second least in all groups at both baseline and follow-up. The average change in access to Serene was insignificant (see Table 2).

Among the movers 165 individuals (11.6%) belonged to “remedy”, *i.e.*, had improved from poor to good mental health. In the “remedy” group of the movers, 17.8% had gained access to Serene, while 8.0% had lost access. Among those belonging to “sicken” (11.4%) 6.9% had gained access and 14.0% had lost access to Serene. Among the “remainers” 10.1% belonged to “remedy”.

**Table 1:** Mental health, demographics, and amount of nature qualities in the three groups: (1) “movers” (n = 1419); (2) “remainers” (n = 7811); and (3) “all” (n = 9230) at baseline and follow-up. Available data on gender and age for non-responders 2005 are also presented.

Variables	1999 Movers	1999 Remainers	1999 All	2005 Movers	2005 Remainers	2005 All	Non Responders (2005)
Poor mental health (%)	20.6	15.5	16.4	21.0	14.9	15.8	
No. of qualities (µ) *	1.0	0.66	0.72	0.98	0.66	0.71	
Age	42.7	51.,4	50.1	47.7	56.4	55.1	51.8
Female (%)	57.6	55.0	55.4	57.6	55.0	55.4	49.7
Unmarried (%)	36.2	23.3	25.3	29.7	24.5	25.3	
Edu. level (%):							
<10 y	23.2	36.2	34.2	22.6	36.4	34.2	
10–12 y	38.6	28.6	30.2	31.1	26.2	27.0	
vocational	11.5	10.0	10.2	11.7	9.5	9.8	
university	26.7	25.2	25.4	34.7	27.9	29.0	

Note: * the mean value of nature qualities refer to an average of the number of qualities that residents in the respective group had access to within the buffer zone.

**Table 2:** Percentage of the population with access to each quality respectively (within 300 m) at baseline and at follow-up among “movers” and “all”. Baseline values for remainers (no change in values over time).

Quality	Movers		Remainers	All	
Access to:	1999 (%)	2005 (%)	1999 (%)	1999 (%)	2005 (%)
Wild	5.4	5.0	3.2	3.6	3.6
Space	18.0	13.8	9.9	11.2	10.5
Serene	9.5	9.1	5.6	6.2	6.1
Culture	34.2	38.1	22.1	24.0	24.8
Lush	36.2	32.3	25.1	26.9	26.1

The odds for improved mental health (being a “remedy”) were significantly higher among those that got increased access to the quality Serene (see Table 3).

**Table 3:** Odds ratios for improved mental health (remedy) by gained access to a nature quality.

Gained Access to:	Odds Ratio (OR)	CI
Serene	2.80	1.11–7.04
Lush	1.18	0.71–1.95
Culture	1.37	0.86–2.20
Space	1.35	0.65–2.80
Wild	1.28	0.42–3.89

There was a significant correlation (chi-square *p* = 0.045, linear-by linear association and Pearson’s R *p* = 0.013) between *change* in mental health (delta-value) and change in the quality Serene between baseline and follow-up. Without change in this quality no change in mental health was demonstrated.

Regarding research question number one we found no significant association between change of *amount* of nature qualities and change in mental health.

Regarding research question number two we found a significant correlation between gaining access to the nature quality Serene and recovering from poor to good mental health in 2005. The incidence of poor mental health was 13.1% in the group with access to serenity and 21.4% in the group with no access. No significant correlations were found for the other nature qualities nor for combinations.

Logistic regressions for men and women (*n* = 717) separately (improved mental health, “remedy”, as dependent variable) demonstrated a significant correlation between improved mental health among *women* and gained access to the quality Serene, adjusted for age, economy, experienced stress, ethnicity, and education. The chance for better mental health without any change in the environment was expressed with the odds 2.7:1, as compared to losing access to Serene. By gaining access to Serene, the odds for remedy was 4.5:1, compared to losing access. The probability for getting better mental health was 82% $(P=\exp\left(B_{0}+B_{1}X_{1}+B_{2}X_{2}+B_{3}X_{3}\right)/\left(1+\text{exp}\left(B_{0}+B_{1}X_{1}+B_{2}X_{2}+B_{3}X_{3}\right)\right)$ (Table 4).

**Table 4:** Chance of getting improved mental health (dependent variable) among women in relation to the categorical exposure variable “access to Serene” and the other significant independent variables age and economy (*n* = 717).

Coefficients	*B*	*p*	Odds Ratio (OR)	CI
Constant	−2.19	0.10	0.11	
Change in Serene ^†^				
No change in Serene	0.99	0.07	2.71	0.92–3.01
Gained Serene	1.51	0.02	4.51	1.29–5.83
Age	−0.03	0.002	0.975	0.96–0.99
Economy	0.60	0.05	1.82	0.99–3.36

^†^ Dummy coded in SPSS, reference = lost access to serene. Test: Cox & Snell’s *R*^2^ = 0.049. Nagelkerkes’s *R*^2^ = 0.088. Hosmer and Lemeshow Chi^2^ = 12.2; *p* = 0.14. Non-significant variables, change in education-level, marital status and self-perceived stress, were not included in the model and are not presented in the table. Neither are the results for men presented, since the model was not approved.

The model for women $(\left(\text{ln}\left(\frac{P}{1-P}\right)=B_{0}+B_{1}X_{1}+B_{2}X_{2}+B_{3}X_{3}\right)\;$explained a significantly (*p* < 0.001) higher proportion of the mental health change than the zero model. The variables change in stress, ethnicity, and education group were not significant and not kept in the model. For men the model was not significant and a final solution could not be found with the selected variables.

## 4. Discussion

The purpose of this study was to evaluate correlations between mental health and amount or type of nature qualities in the neighborhood, and whether any differences were to be found between the sexes. We did not find any significant effect on mental health by moving to an area with an increased number of nature qualities in the neighborhood. However, we found a significant relationship between gained access to the nature quality Serene and improved mental health among women, though the sample was very small. We did not find a significant model for men with the chosen covariates. Apart from Serene, no specific nature quality had any significant impact on mental health.

The strength of our approach is the longitudinal perspective in an initially relatively large cohort. Data were achieved from a broad and extensive health survey, providing good opportunities for confounding adjustments. The mental health score was based on GHQ-12, an internationally tested reliable and validated instrument. We used geographically objective land use data for definitions of the nature qualities, which facilitated neighborhood assessment for each quality for each resident and we could explore mental health values of specific nature qualities.

The original cohort of suburban and rural individuals (*n* = 9230) was large enough to reduce most random errors, but a major limitation with the study is the small number of movers. An even smaller amount experienced any change in mental health and could be included in the multivariate analyses, which severely impairs the possibility for drawing causal conclusions from the results. Another considerable limitation is that we only have data for two consecutive occasions and the absolute significance level of environmental change is unknown. However, the variables in the equation are delta-values, for which we found a significant correlation between the change in mental health and change in the quality Serene. We do not know where in the five years period between the two surveys the individuals actually moved, neither the motives for the move, other related life events, or any anticipation effects. There is also a risk for selection bias due to possible specific traits of people in the moving group.

Regarding the environment, we know that other variables have an impact than nature qualities, such as socioeconomic deprivation. This variable was included on an individual level, but not on the overall area level. We were only able to use five nature qualities out of originally eight, and although developed by experts in landscape planning, the qualities themselves are not yet to be considered as validated constructs*.* However, the qualities that have previously been found most important for stress relief (wild, serene, and space) were included [27].

Due to land use data restrictions for urban areas we studied only suburban and rural areas. However, this reduces the risk for confounding urbanity with nature quality as an effect variable of moving. Our study is also restricted by the geographical constraint, which impairs the possibility to generalize the results. Southern Sweden has a particular landscape, with a relatively uniform composition of agricultural land and deciduous or mixed woodlands. This may explain the generally low amount of neighborhood nature qualities (0.66 among the remainers, and 1.0/0.98, at baseline and follow-up respectively, among the movers). It is plausible that in a more heterogeneous landscape area, including more nature qualities, the effect sizes would have been larger and potentially revealed effects also by amount of or other qualities.

Another limitation is that the Nagelkerke’s *R*^2^ for the logistic regression model was small (0.088), something that is often the case when studying multifactorial interactions [47]. Although the relevance of pseudo-*R*^2^ in logistic regressions has been questioned [48], it indicates that we cannot with the aid of these models with any certain precision predict which individuals will have a positive change in mental health. However, we have shown that some factors may influence the odds of improved mental health to an extent that most probably cannot be explained by random effect, though with the caution of the small sample. The Hosmer and Lemeshow test approved the model, *i.e.*, a significant, but small, proportion of the chance for improved mental health would be explained by the model. The final model for women included only economy and age, apart from access to Serene, and it is possible that we might have neglected other explanatory variables, though we initially adjusted for several common confounders, like education, ethnicity and self-perceived stress.

In relation to previous research on nature and health our results differ slightly. Whereas it has usually been suggested that “the more the merrier” and that access to or levels of green spaces, without further definition, is related to better health [6,49] we did not find this general association, although we found a specific nature quality (Serene) to be of a potentially specific value. Similar kind of qualities have been captured in other studies where people’s motives for visiting nature are often to find dimensions of peacefulness, quietness, calm, and tranquility, [25,36,37,50,51]. Several empirical studies show an apparent relationship between preferences and restorativeness [52,53,54]. According to the definition of Serene, certain aspects like the sound of nature without any disturbing noise and elements of water are important for the experience. This relates, for example, to research on the negative health and stress aspects of noise [55,56] as well as to the suggested positive impact of natural sounds [21,57]. Serene is described to include sounds of winds, birds and insects, which may be interpreted as a certain kind of acoustic biodiversity, and diversity in sounds have in previous research been shown to relate to landscape preference and wellbeing [58,59]. This may be part of the explanation to why Serene may have particular importance for health. Another aspect is the water exposure. Recently the particular effect of blue spaces has gained attention and a few studies have demonstrated distinctly positive health impact of access to water [60,61]. This is also in accordance with evolutionary theories on nature’s inherent positive health impact; water is considered a crucial element for human survival and thus evokes an immediate feeling of wellbeing and stress relief [62]. In addition, water is seen as a particularly non-demanding feature and would thus suit people in stress or crisis with a certain need for restorative environments [63].

The study indicates that there might be a difference between how women and men may benefit from surrounding nature qualities. If this is so, many mechanisms behind this phenomenon may be discussed, such as varied use of nature, varied stress responses and mental disorder aetiology. It might also be that there exists a gender difference of perceiving and experiencing natural landscapes, something that has been suggested by brain-imaging studies demonstrating gender-related differences in the neural correlates of aesthetic preference [64]. Gender differences have also been demonstrated in a few other studies on relationships between green spaces and health [49,65]. The plausible gender difference in perceiving nature, and in particular the quality Serene*,* and the relation to health should be further explored.

## 5. Conclusions

This study proposes that serene nature may prevent mental illness, especially among women. By exploring this further it could contribute to healthy urban planning, advising undisturbed and safe environments with silence, no noise or litter (noise over 30 dB or presence of artillery range were exclusion criteria for Serene in the objective neighborhood GIS analysis). It would also be recommended to include broadleaved trees and water elements in urban green spaces to potentially increase the positive health effects of access to nature. Notwithstanding some limitations and restrictions we believe that this piece of evidence may bring us closer to efficient use of green spaces as a public health resource.
